# Isoprene Emission in Darkness by a Facultative Heterotrophic Green Alga

**DOI:** 10.3389/fpls.2020.598786

**Published:** 2020-11-11

**Authors:** K. G. Srikanta Dani, Giuseppe Torzillo, Marco Michelozzi, Rita Baraldi, Francesco Loreto

**Affiliations:** ^1^ Institute for Sustainable Plant Protection, National Research Council of Italy, Florence, Italy; ^2^ Department of Biology, Agriculture and Food Sciences, National Research Council of Italy, Rome, Italy; ^3^ Institute of Bioeconomy, National Research Council of Italy, Florence, Italy; ^4^ Institute for Biosciences and Bioresources, National Research Council of Italy, Florence, Italy; ^5^ Institute of Bioeconomy, National Research Council of Italy, Bologna, Italy; ^6^ Department of Biology, University Federico II, Naples, Italy

**Keywords:** *Chlorella vulgaris*, chlorophyll, volatile hydrocarbons, glycolysis, photosynthesis in unicellular eukaryotes, heterotrophy, marine isoprene

## Abstract

Isoprene is a highly reactive biogenic volatile hydrocarbon that strongly influences atmospheric oxidation chemistry and secondary organic aerosol budget. Many phytoplanktons emit isoprene like terrestrial pants. Planktonic isoprene emission is stimulated by light and heat and is seemingly dependent on photosynthesis, as in higher plants. However, prominent isoprene-emitting phytoplanktons are known to survive also as mixotrophs and heterotrophs. *Chlorella vulgaris* strain G-120, a unicellular green alga capable of both photoautotrophic and heterotrophic growth, was examined for isoprene emission using GC-MS and real-time PTR-MS in light (+CO_2_) and in darkness (+glucose). *Chlorella* emitted isoprene at the same rate both as a photoautotroph under light, and as an exclusive heterotroph while feeding on exogenous glucose in complete darkness. By implication, isoprene synthesis in eukaryotic phytoplankton can be fully supported by glycolytic pathways in absence of photosynthesis, which is not the case in higher plants. Isoprene emission by chlorophyll-depleted mixotrophs and heterotrophs in darkness serves unknown functions and may contribute to anomalies in oceanic isoprene estimates.

## Introduction

Isoprene, the most prominent of all biogenic volatile organic compounds (BVOCs) in the atmosphere, influences atmospheric oxidation status and secondary aerosol yield over terrestrial ecosystems globally, and over marine emission hotspots locally (Henze et al., 2008; Wang and Ruiz, 2017). Isoprene emission from plant leaves is strictly light-dependent and although metabolic intermediates from glycolysis augment photosynthetic carbon used for isoprene synthesis, the emission from leaves ceases quickly in darkness (Loreto and Sharkey, 1990; Schnitzler et al., 2004; Fasbender et al., 2018). Some bacteria emit isoprene without photosynthesis (Wagner et al., 2000), and when chemical energy is not limiting, isoprene can be arguably synthesized in presence of any carbon source (Dani et al., 2015). Past studies had detected tiny amounts of isoprene in the static headspace of thalloid algae and phytoplankton after many days of dark-incubation (Broadgate et al., 2004; Ooki et al., 2019). Yet, light- and heat-responsive isoprene emissions have been the norm for known isoprene-emitting phytoplankton (Exton et al., 2013; Dani et al., 2017). Field-based estimations of isoprene from marine and freshwater sources routinely rely on correlations between chlorophyll abundance, photosynthesis, and species distribution (Hackenberg et al., 2017; Booge et al., 2018; Steinke et al., 2018; Rodríguez-Ros et al., 2020). However, many prominent marine isoprene emitters, such as dinoflagellates and coccolithophores, are facultative heterotrophs and mixotrophs (Johnson, 2015; Dani and Loreto, 2017). Mixotrophic and heterotrophic growths often lead to chlorophyll depletion in such organisms (Terrado et al., 2017), and the presumed equations among chlorophyll abundance, photosynthesis, species distribution, and isoprene emission in mixed-ocean layers become tenuous. It was therefore important to verify if a unicellular eukaryote could emit isoprene in absence of photosynthesis.

Marine heterotrophic protists are not easy to culture especially at high cell densities, and their metabolic pathways are scarcely investigated. *Chlorella* is a unicellular photoautotrophic freshwater green alga that has been a model for research on photosynthesis and glycolysis for several decades. *Chlorella* has simple nutrient media needs, shows quick doubling time (~8 h) as a photoautotroph utilizing dissolved CO_2_ and HCO_3_^−^, and more importantly some strains can become heterotrophic in presence of exogenous sugars (Beardall and Raven, 1981; Masojidek and Torzillo, 2008). *Chlorella* is also a suitable model to examine planktonic isoprene emission since it lacks the cytosolic mevalonic acid pathway for isoprenoid synthesis and possesses only the plastid-localized (prokaryotic) methylerythritol phosphate (MEP) pathway (Disch et al., 1998), the source of isoprene in all emitting organisms. Using a fast-growing facultative heterotrophic strain of *Chlorella vulgaris*, we tested if *Chlorella* emits isoprene while growing heterotrophically exclusively on glucose in the dark.

## Materials and Methods

### Chlorella Culturing and Maintenance

An axenic stock of *C. vulgaris* strain G-120 (Chlorophyceae) was assessed as a competent heterotroph feeding on glucose in complete darkness (Babaei et al., 2020). Before starting the experiments, stock cultures were plated on organic medium (glucose, 3 g L^−1^; yeast extract; 3 g L^−1^; peptone, 3 g L^−1^ and with agar 12 g L^−1^; Torzillo et al., 1985). An inoculum was prepared in mineral medium containing KNO_3_ (1.71 g L^−1^), K_2_HPO_4_ (0.42 g L^−1^), MgSO_4_ (0.27 g L^−1^), with microelements and Fe-EDTA in 250 ml Erlenmeyer flasks placed within an illuminated orbital shaker-incubator. This inoculum was transferred to vertical glass screw-cap columns containing 200 ml fresh medium and incubated at 30 ± 0.5°C in a circulating water bath under continuous cool fluorescent light (150 μmol photons m^−2^ s^−1^; Dulux L, 55 W/840, Osram, Italy) and bubbled constantly with filtered mixture of compressed air-CO_2_ (97/3, v/v from a gas cylinder with flow-control). Culture was replenished by replacing 30% of volume by fresh medium once every 3 days and several 400 ml photoautotrophic cultures were generated. An axenic sample from the primary stock (photoautotrophic) was used to prepare a 50 ml inoculum in a glucose-enriched medium (glucose, 10 g L^−1^; KNO_3_, 12 g L^−1^; K_2_HPO_4_, 2 g L^−1^; MgSO_4_, 3.3 g L^−1^), with sterilized microelements added separately; pH = 6.2). The inoculum generated for heterotrophic growth was supplied with a mixture of antibiotics (Penicillin G, 50 mg L^−1^ and Streptomycin sulfate, 30 mg L^−1^) to further ensure bacteria-free growth in presence of glucose. Culture was raised in a 250 ml Erlenmeyer flask incubated at 30°C for 7 days in darkness and then scaled-up to 400 ml (fed with air-CO_2_) for measurements in darkness. All cultures were maintained under optimal conditions and monitored microscopically to ensure absence of bacterial contamination. Chlorophyll content and culture dry weight were estimated by following Dani et al. (2017), and were quantified within ±1 day of isoprene sampling. Since by design, there was only one possible biological replicate (i.e., the master stock of *C. vulgaris* G-120), these working cultures were raised afresh from the parental stock multiple times to achieve technical replication of experiments.

### Isoprene Quantification by GC-MS and PTR-MS

VOC-free air, generated using a portable zero-air generator (Parker Chromgas 1,000, Supelco, USA), was supplied to 400 ml cultures, and maintained at 30 ± 1°C in cylindrical tubes, by bubbling air at 200 ml min^−1^ through the culture. The air outflow was split and a sub-sample was adsorbed onto Carbotrap (Supelco, Sigma-Aldrich) adsorbent beds packed in perforated thermal desorption tubes, using a mass flow-controlled pump. Isoprene was quantified *via* TD-GC-MS (thermal desorption-gas chromatography-mass spectrometry) by an Agilent 7890 gas chromatograph equipped with a 5975-mass spectrometer (Agilent Technologies, CA, USA; after Dani et al., 2017). Experiments were technically replicated by raising fresh cultures (6–10 independent replicates per treatment). Photoautotrophic cultures were provided with 150 μmol photons m^−2^ s^−1^ while heterotrophic cultures were kept in darkness. Blank mineral medium and blank glucose medium (with antibiotics) were used as control. Readings from mineral and glucose blank medium were subtracted from total isoprene detected from cultures to obtain actual isoprene emission rate attributed to *Chlorella*.

Real-time emission was monitored using a Proton Transfer Reaction-Mass Spectrometry (PTR-MS; Ionicon, Analytic GmbH, Innsbruck, Austria), VOC-free air generated from a clean air generator (comprising platinum catalyst pellets heated to 380°C) was supplied to the culture at 50 ml min^−1^. The outflow was split to collect a subsample at the PTR-MS inlet set to 60°C. The drift tube pressure was 2.2mbar, an electrical field strength was 600 V cm^−2^,and an ionization energy E/N = 130 Td was applied, which yielded ~4 × 10^7^ ion counts per second (cps) for primary H_3_O^+^ ions. Protonated isoprene (^12^C_5_H_8_^H+^; *m*/*z* 69) and its naturally occurring isotopomer (^13^C_1_^12^C_4_H_8_^H+^; *m*/*z* 70) were monitored under Selected Ion Monitoring (SIM) mode. To test whether photosynthetic and/or glycolytic carbon were used for isoprene synthesis in light, ^13^C-bicarbonate (1.2% w/v of NaH^13^CO_3_, Sigma-Aldrich, USA) and ^12^C-glucose (3% w/v) were fed to photoautotrophic *Chlorella* (see section Supplementary Material for details). The change in abundance of protonated unlabeled isoprene (*m*/*z* 69), partially labeled (*m*/*z* 70, 71, 72), and fully labeled isoprene (*m*/*z* 74) were monitored PTR-MS. The results in cps were converted to nmol s^−1^ with calibration curves from an isoprene standard mixture.

### Statistical Analysis

After testing for normality (Anderson-Darling test) and equality of variance (*F* test), the significance of differences in emission rate and in chlorophyll content between phototrophic (light) and heterotrophic cultures (dark) was tested by student’s *t* test (*α* = 0.05, *N* = 6–10). Statistical tests were carried out using Minitab v19 stats package (Minitab Inc., USA).

## Results

**Figure 1:**
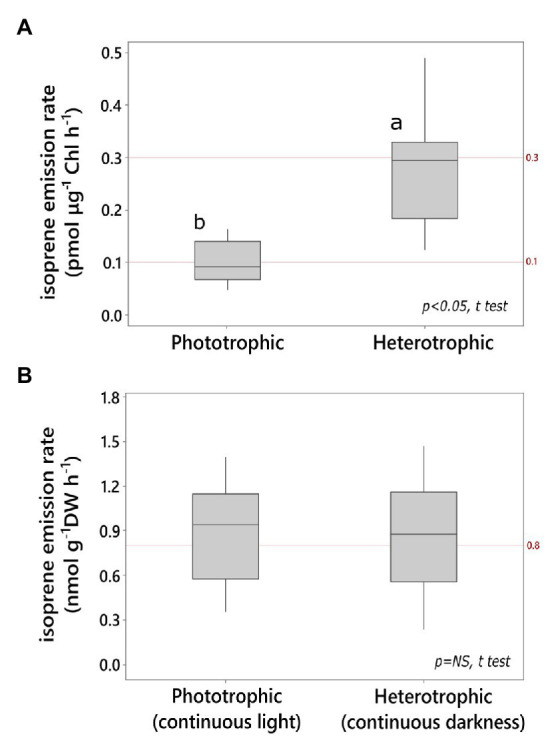
Light and dark isoprene emission by *Chlorella vulgaris* **(A)** on chlorophyll (Chl) basis, **(B)** on culture dry weight (DW) basis. Emission in the light was measured from phototrophic cultures (minimal mineral medium). Emission in the dark was measured from heterotrophic cultures (glucose medium; *N* ≥ 6 technical replicates, *t* test, *α* = 0.05).

Isoprene emission rate by heterotrophic cultures in darkness was significantly higher per unit chlorophyll content compared to that by phototrophic cultures in light (Figure 1A). The heterotrophic cultures grown on glucose in darkness contained 0.42 ± 0.06% Chl DW^−1^ after up to 6 days of dark acclimation, and they were significantly chlorophyll-depleted compared to light-grown photoautotrophic cultures (2.6 ± 0.3% Chl DW^−1^; *N* = 6 independent cultures each, *p* < 0.05; *t* test). Cell density in heterotrophic *Chlorella* (8.7 ± 0.7 g DW L^−1^; ~0.3 × 10^9^ cells ml^−1^) was 30% higher than that in phototrophic cultures grown on mineral medium in the light (6.1 ± 0.8 g DW L^−1^, ~0.2 × 10^9^ cells mL^−1^; *N* = 6 independent cultures each; *p* < 0.05; *t* test). However, both phototrophic and heterotrophic *Chlorella* emitted isoprene at comparable levels on a biomass basis (Figure 1B). ^13^C-bicarbonate feeding led to a significant increase in the relative abundance of fully labeled isotopomer (^13^C_5_H_8_^H+^, *m*/*z* 74) and a significant decrease in the fully unlabeled isotopomer of isoprene (^12^C_5_H_8_^H+^, *m*/*z* 69; Supplementary Figure S1). Addition of ^12^C-glucose (3% w/v) to this culture (prelabeled with ^13^C-bicarbonate) led to immediate increase in the partially labeled isotopomer (^13^C_2_^12^C_3_H_8_^H+^; *m*/*z* 71) followed by a lagged increase in partially labeled isotopomer of isoprene (^13^C_3_^12^C_2_H_8_^H+^; *m*/*z* 72; Supplementary Figure S1).

## Discussion

Light-independent isoprene synthesis and emission are unknown in higher plants. This is the first demonstration of a photoautotrophic eukaryote emitting isoprene as a facultative heterotroph in the dark.

**Figure 2:**
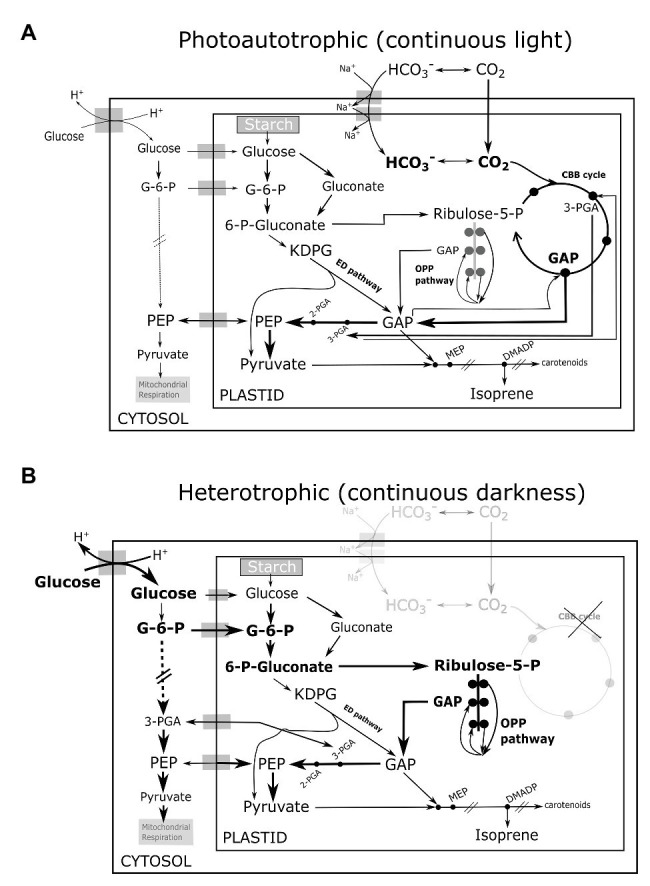
Illustration of proposed interactions among glycolytic pathways, photosynthesis, and isoprene biosynthesis in **(A)** photoautotrophic eukaryotes in continous light **(B)** heterotrophic eukaryotes in continuous darkness. Metabolic cross-talk among CBB cycle (active only in light), the three glycolytic pathways namely the OPP pathway, the ED pathway, and cytosolic glysolysis, is indicated by prominent arrows. The thickness of arrows indicate the potential flux strength. The CBB cycle and glycolytic pathways co-contribute GAP, pyruvate to the MEP pathway and isoprene synthesis in light. The three glycolytic pathways sustain carbon and energy supply for isoprene synthesis in darkness. CBB, Calvin-Benson-Bassham; OPP, oxidative pentose phosphate; G-6-P, glucose-6-phosphate; ED, Entner-Doudoroff; 6-P-gluconate, 6-phosphogluconate; KDPG, 2-keto-3-deoxy-6-phosphogluconate; Ribulose-5-P, ribulose-5-phosphate; 3-PGA, 3-phosphoglycerate; 2-PGA, 2-phosphoglycerate; GAP, glyceraldehyde-3-phosphate; PEP, 2-phosphoenol pyruvate; MEP, 2-C-methyl-D-erythritol 4-phosphate; and DMADP, dimethylallyl diphosphate.

Incontrovertibly, glycolysis is the primary source of carbon and energy for all anabolic processes including isoprene synthesis in heterotrophic *Chlorella* growing in darkness. Pioneering experiments in algae had traced carbon atoms from exogenous glucose in MEP-derived phytols (chlorophyll side-chains) and carotenoids in low-light (Disch et al., 1998), and our results show that even isoprene can be synthesized in darkness. Some steps of the glycolytic pathway are mostly or solely active in the chloroplasts of planktonic green algae (Klein, 1986; Schnarrenberger et al., 1994). The plastidial oxidative pentose phosphate (OPP) glycolytic pathway (the sole source of NADPH in darkness) is likely as important as cytosolic glycolysis (Yang et al., 2000; Xiong et al., 2010; Fan et al., 2015) in generating metabolites for isoprene emitted in darkness. The Entner-Doudoroff (ED) glycolytic pathway sustains the MEP pathway in bacteria (Rohmer et al., 1996; Liu et al., 2013) and the same can support dark isoprene synthesis in heterotrophic eukaryotes (Figures 2A,B). A diagnostic test involving NaH^13^CO_3_ feeding of photoautotrophic culture under light indeed showed slow and incomplete inclusion of ^13^C in isoprene, whereas subsequent addition of ^12^C-glucose quickly unlabeled a C3 moiety of isoprene molecule reflected in immediate increase in the abundance of partially labeled isotopomer ^13^C_2_^12^C_3_H_8_^H+^ (*m*/*z* 71; see Supplementary Table 1). This was followed by a delayed increase in ^13^C_3_^12^C_2_H_8_^H+^ (*m*/*z* 72; Supplementary Figure S1), and suggested that C2-fragments from glycolytic pyruvate enter isoprene first, while C3-fragments are sourced mainly from photosynthetic GAP similar to plant leaves (Trowbridge et al., 2012). Overall, we postulate that planktonic isoprene emission is more dependent on glycolytic pathways than on photosynthesis, which is the opposite of what we know in higher plants. Assuming that *Chlorella* and eukaryotes in general do not use an exotic mode other than the MEP pathway to emit isoprene, further experiments using ^13^C-glucose and ^13^C-labeled glycolytic intermediates (fed) in the dark will reveal the relative importance of various glycolytic pathways (Figure 2B) to isoprene synthesis in mixotrophic and heterotrophic phytoplankton.

Much of global oceans and freshwater bodies are oligotrophic, and those habitats may not support dark emission of isoprene at the same rates observed in a nutrient-rich laboratory culture of a fast-growing model green alga. However, the capacity for mixotrophy *via* importing of exogenous sugar exists even in isoprene-emitting photoautotrophic cyanobacteria (Muñoz-Marín et al., 2013), which suggests that isoprene can be synthesized in darkness by potentially all photoautotrophic phytoplankton. Non-photosynthetic phagotrophs may benefit (attract bacteria) from isoprene made in the dark, although relevance of dark emission in *Chlorella*, which is photosynthetic by default, deserves better answers. Isoprene degradation and consumption by bacteria (McGenity et al., 2018), nutrient availability (Zindler et al., 2014), and variable water-to-air efflux of isoprene are acknowledged as important correctives for chlorophyll-based estimations of oceanic isoprene. Our results highlight that a fourth factor, i.e., heterotrophic emission in light and in darkness may weaken an otherwise positive correlation between primary productivity and isoprene concentrations in the seas.

## Data Availability Statement

The raw data supporting the conclusions of this article will be made available by the authors, without undue reservation.

## Author Contributions

KGSD designed and conducted the experiments with inputs from GT and FL, who first proposed the study. MM and RB assisted. KGSD analyzed results, and wrote and revised the manuscript with edits from GT and FL. All authors contributed to the article and approved the submitted version.

### Conflict of Interest

The authors declare that the research was conducted in the absence of any commercial or financial relationships that could be construed as a potential conflict of interest.
