# Supplementary material for: Isoprene Emission in Darkness by a Facultative Heterotrophic Green Alga
Source: Front Plant Sci. 2020 Nov 11;11:598786. doi: 10.3389/fpls.2020.598786 (PMC7686029; doi:10.3389/fpls.2020.598786)
Supplement: Supplementary file 1 [file Data_Sheet_1.docx]

**ORIGINAL RESEARCH ARTICLE**

Front. Plant Sci. | doi: [10.3389/fpls.2020.598786](https://doi.org/10.3389/fpls.2020.598786)

**Isoprene Emission in Darkness by a Facultative Heterotrophic Green Alga**

***K. G. Srikanta Dani ^1,2*^, Giuseppe Torzillo ^3^, Marco Michelozzi ^4^, Rita Baraldi ^5^ and Francesco Loreto ^2,6*^***

^1^Institute for Sustainable Plant Protection, National Research Council of Italy, Florence, Italy, ^2^Department of Biology, Agriculture and Food Sciences, National Research Council of Italy, Rome, Italy, ^3^Institute of Bioeconomy, National Research Council of Italy, Florence, Italy, ^4^Institute for Biosciences and Bioresources, National Research Council of Italy, Florence, Italy, ^5^Institute of Bioeconomy, National Research Council of Italy, Bologna, Italy, ^6^Department of Biology, University Federico II, Naples, Italy

Supplementary Material

# Supplementary methods

- 1. ***^13^C bicarbonate feeding and real time monitoring by PTR-MS***

To test if carbon from photosynthetic intermediates and exogenous glucose is used for isoprene synthesis, a diagnostic test using ^13^C-bicarbonate and ^12^C-glucose feeding was performed usingphotoautotrophic *Chlorella* cultures acclimated to 300 μmol photons m^-2^ s^-1^ and 29 ± 1 °C. The feeding trial was conducted twice and the second trail is described here. A 250 mL photoautotrophic culture (5 g DW L^-1^) was centrifuged and resuspended in 100 mL of fresh medium and from this 30 mL was transferred to a 50 mL cylindrical tube connected with flow stoppers on air inlet and outlet. Fully unlabelled (*m/z* 69), partially labelled (*m/z* 70, 71, 72), and fully labelled isoprene(*m/z* 74) were monitored by PTR-MS for 15 min. The *m/z* 73 was excluded due to high background noise possibly by a protonated water-cluster. After monitoring for 15 min, 1.2% w/v of NaH^13^CO_3_ (^13^C 98% by atom, Sigma-Aldrich, USA) was dissolved directly in the culture (pH was 6.9). It takes ~15 min of ^13^CO_2_ feeding to label 85% of isoprene emitted by plant leaves in light. Since our trial involved much slower aqueous feeding, ^13^C-bicarbonate was fed for 80 min. Air flow was stopped 4 times (12 min × 3 + 24 min × 1) to impose static incubation, and labelling was minitored (for 3, 6, 8, 10 min × 1 each cycle) immediately after each stop phase. After 80 min of^13^C-bicarbonate feeding in light, to the same culture 3% w/v of ^12^C-glucose (>99% purity) was added and ion monitoring continued for 20 min in light. It was assumed that the changes in proportions of unlabelled and partially-labelled ion fractions *m/z* 69, 70, 71, 72 were entirely attributable to isoprene emitted by *Chlorella* and were neither artefacts nor unknown byproducts of glucose in the medium.

The relative abundance of individual isotopomers of isoprene before, during, and at the end of ^13^C-bicarbonate feeding (1.2% w/v) and ^12^C-glucose feeding (3% w/v) is given in Table S1. The abundance of *m/z* 74 (^13^C_5_H_8_^H+^) increased from 4% of total before labelling to 18% of total after 80 min of incubation with ^13^C-bicarbonate. Addition of ^12^C-glucose (3% w/v) to this culture led to an immediate increase in partially labelled *m/z* 71 (^13^C_2_^12^C_3_H_8_^H+^) and a slightly delayed increase in *m/z* 72 (^13^C_3_^12^C_2_H_8_^H+^)and the PTR-MS traces are depicted in Figure S1.

| **Table S1: Change in relative abundance of isoprene isotopomers during ^13^C-bicarbonate feeding trial** | | | | | | |
| --- | --- | --- | --- | --- | --- | --- |
| **Time (min)** | **Labelling status** | **Relative abundance (% of total, excluding *m/z* 73)** | | | | |
|  |  | ***m/z* 69** | ***m/z* 70** | ***m/z* 71** | ***m/z* 72** | ***m/z* 74** |
| 0 | Before ^13^C-bicarbonate feeding | 79% | 8% | 5% | 4% | 4% |
| 80 | After 80 min of ^13^C-bicarbonate feeding | 32% | 11% | 30% | 9% | 18% |
| 90 | 10 min after adding ^12^C-glucose | 28% | 9% | 42% | 8% | 13% |
| 100 | 20 min after adding ^12^C-glucose | 20% | 7% | 56% | 8% | 8% |

## Supplementary Figures

**Supplementary Figure 1.**

**Realtime changes in isoprene emission rate during ^13^C-bicarbonate followed by ^12^C-glucose feeding to phototrophic *Chlorella*in light.** Protonated molecular ions of isoprene representing unlabelled (*m/z* 69), partially labelled (*m/z* 70, 71, 72), and fully labelled (*m/z* 74) are shown. Steady-state isoprene emission rate for 3 min prior to labelling is shown. ^13^C-bicarbonate was added and the changes in ion fractions were monitored. The labelling had 4 periods of static and closed head-space incubation (12 min × 3; 24 min × 1) and 4 phasesof PTR-MS monitoring of variable duration (3, 6, 8, 10 min × 1 each). Changes in relative abunfances of isoprene isotopomers are given in Table S1.


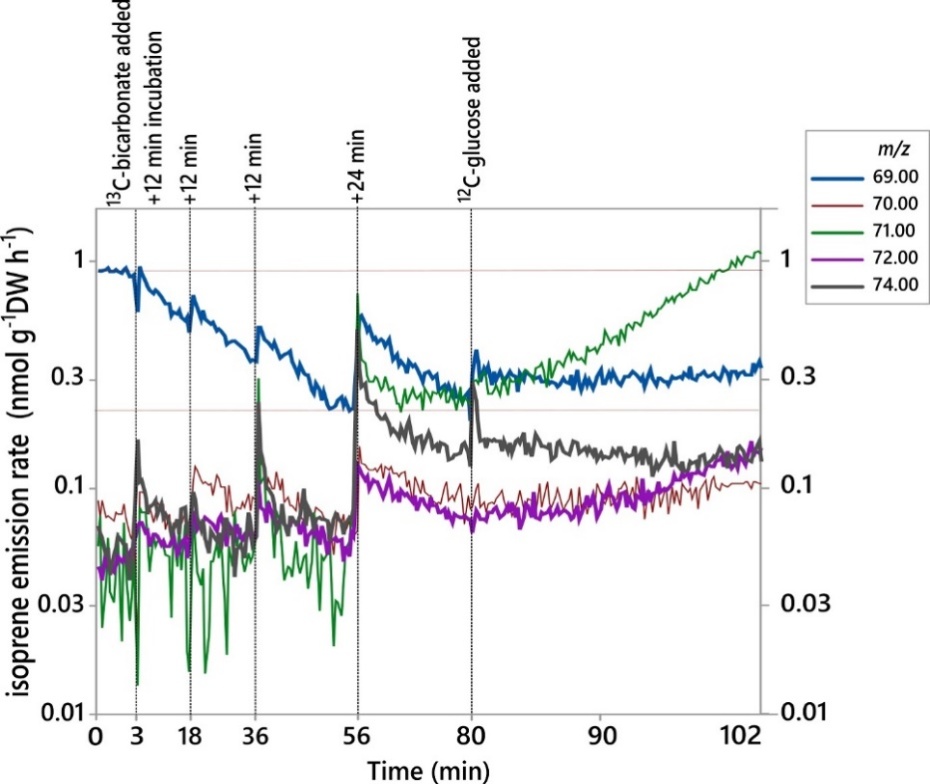


**
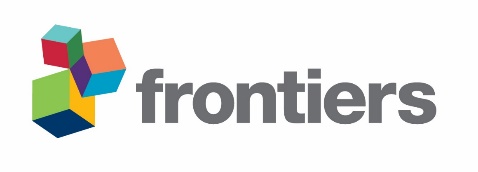
**
